# Hierarchical Development of Motile Polarity in Durotactic Cells Just Crossing an Elasticity Boundary

**DOI:** 10.1247/csf.19040

**Published:** 2019-12-27

**Authors:** Thasaneeya Kuboki, Hiroyuki Ebata, Tomoki Matsuda, Yoshiyuki Arai, Takeharu Nagai, Satoru Kidoaki

**Affiliations:** 1 Laboratory of Biomedical and Biophysical Chemistry, Institute for Materials Chemistry and Engineering, Kyushu University, 744 Moto-oka, Nishi ku, Fukuoka, Japan; 2 Department of Biomolecular Science and Engineering. The Institute of Scientific and Industrial Research, Osaka University, Mihogaoka 8-1, Ibaraki, Osaka, Japan

**Keywords:** Microelasticity patterned gel, durotaxis, cell polarity, focal adhesions, paxillin

## Abstract

Cellular durotaxis has been extensively studied in the field of mechanobiology. In principle, asymmetric mechanical field of a stiffness gradient generates motile polarity in a cell, which is a driving factor of durotaxis. However, the actual process by which the motile polarity in durotaxis develops is still unclear. In this study, to clarify the details of the kinetics of the development of durotactic polarity, we investigated the dynamics of both cell-shaping and the microscopic turnover of focal adhesions (FAs) for Venus-paxillin-expressing fibroblasts just crossing an elasticity boundary prepared on microelastically patterned gels. The Fourier mode analysis of cell-shaping based on a persistent random deformation model revealed that motile polarity at a cell-body scale was established within the first few hours after the leading edges of a moving cell passed through the boundary from the soft to the stiff regions. A fluorescence recovery after photobleaching (FRAP) analysis showed that the mobile fractions of paxillin at FAs in the anterior part of the cells exhibited an asymmetric increase within several tens of minutes after cells entered the stiff region. The results demonstrated that motile polarity in durotactic cells is established through the hierarchical step-wise development of different types of asymmetricity in the kinetics of FAs activity and cell-shaping with a several-hour time lag.

## Introduction

Directional cell movement, so-called cellular taxis, is an essential phenomenon in living organisms, and is observed not only in normal physiological processes such as embryo development and wound healing (Martin, 1997; Keller, 2005), but also in pathological conditions such as cancer metastasis (Wang *et al.*, 2005). In general, the driving factors of taxis are asymmetric cues consisting of various kinds of tactic attractants or repellants in the extracellular environment such as gradients of chemicals, stiffness, electric current, light, gravity, etc. Among these inducers of taxis, a stiffness gradient in the cell culture matrix leads cells toward the stiffer region of the substrate, which is known as durotaxis (Lo *et al.*, 2000). Durotaxis is an important form of taxis for studies on the mechanics of substrate-adhesion-based cell movement, i.e., cell crawling, as well as several developmental processes and the progression of pathological diseases (Paszek *et al.*, 2005; Ulrich *et al.*, 2009; Tse and Engler, 2011; Choi *et al.*, 2012).

To better understand the mechanics of cell crawling, extensive studies have been performed on the cellular responses to mechanical stimuli that affect cell motility (Pelham and Wang, 1997; Tan *et al.*, 2003; Jiang *et al.*, 2006). To date, the molecular mechanisms of substrate-stiffness-dependent regulation of cell migration have been investigated for cells randomly crawling on substrates with a uniform stiffness. The data suggested the involvement of complicated signaling cascades that regulate the movement machinery of cells such as integrin engagement, dynamics of focal adhesion (FA), cytoskeletal reorganization and force transmission (Prager-Khoutorsky *et al.*, 2011). Though recent studies have tried to elucidate the factors that induce durotaxis (Plotnikov *et al.*, 2012; Plotnikov and Waterman, 2013; Wong *et al.*, 2014; Wormer *et al.*, 2014), the essential problem has remained largely unexplored; how does a stiffness gradient generate and maintain motile polarity in a durotactic crawling cell? To elucidate this issue, single cells just crossing a well-defined stiffness gradient boundary should be analyzed in detail in terms of both the behavior of cell-shaping and the microscopic molecular behavior of the movement machinery.

In this study, to explore the kinetic process for the development of motile polarity in durotactic cells, we characterized asymmetric kinetics in both dynamics of cell-shaping and paxillin activity at FAs for Venus-paxillin-expressing 3T3 fibroblasts just crossing an elasticity boundary between soft and stiff regions. FAs are potential candidates responsible for cellular mechanosensitivity (Wang *et al.*, 2001; Plotnikov *et al.*, 2012). The greater extent of FA formation in the stiff region is believed to be the driving factor for durotaxis (Pelham and Wang, 1997; Beningo *et al.*, 2001). Within FA complexes, paxillin is one of the major scaffolding proteins that contributes to the binding and recruitment of several FA proteins (Deakin and Turner, 2008). A previous study suggested that paxillin likely plays a role in directional cell migration in response to physical cues (Sero *et al.*, 2012).

To prepare a well-defined elasticity boundary, photolithographic microelasticity patterning of photocurable gelatin was used (Kidoaki and Matsuda, 2008; Kawano and Kidoaki, 2011; Kidoaki and Sakashita, 2013; Kuboki *et al.*, 2014; Ueki and Kidoaki, 2015). The precise timing of the generation of cellular polarity when a cell crossed the boundary was quantified with a Fourier mode analysis of the cell shape (persistent random deformation (PRD) model (Ebata *et al.*, 2018)), which makes it possible to precisely characterize the cell-shaping dynamics. On the other hand, the microscopic asymmetric activity in the turnover of FAs was characterized in the anterior part of durotactic cells using fluorescence recovery after photobleaching (FRAP). Based on these dual analyses for shaping dynamics and FA activity in durotactic cells, here we clarify the impact of asymmetric mechanical stimuli arising from the stiffness gradient on these responses in cell polarity generation with hierarchical different time scales.

## Methods

### Cell culture

Mouse fibroblasts (NIH/3T3, Health Sciences Research Resource Bank, Osaka, Japan) in passage 4 were cultured in Dulbecco’s modified Eagle’s medium (DMEM) (Gibco BRL, Grand Island, NY, USA) supplemented with 10% fetal bovine serum (FBS) (Gibco BRL), 100 units/mL penicillin, and 100 μg/mL streptomycin. Cells were maintained on tissue culture polystyrene dishes at 37°C under 5% CO_2_ in a humidified incubator.

### Cloning of paxillin into the N-terminal of pEF-Venus and stable transfection of Venus-paxillin into 3T3 fibroblasts

The full length paxillin was fused in frame with the C-terminal of the yellow fluorescent protein Venus under the control of EF-1alpha promoter (Supplementary method). The construct with correct sequences was used for transfection into 3T3 fibroblasts using Lipofectamine LTX (Life Technology, Tokyo, Japan), according to the manufacturer’s instructions. Stable transfection was performed using culture media with 500 μg/ml of G418. After one week, the isolated colonies were picked up and transferred to a 96-well glass base plate. Positive colonies with bright fluorescence and correct localization of paxillin were further expanded and maintained in DMEM with 250 μg/ml G418.

### Fabrication of microelastically-patterned gels with a square stiff domain

Microelastically-patterned gels with a square stiff domain (400×400 μm) were fabricated as previously described using a custom-built, mask-free photolithography system (Itoga *et al.*, 2004; Kuboki *et al.*, 2014; Ueki and Kidoaki, 2015). Twenty-five microliters of sol solution of 30 wt% styrenated gelatin was sandwiched between two cover glasses (18 mmφ), and first irradiated for 60–90 s to generate the soft base gel. Next, the stiff domain was irradiated for an additional 180–200 s through masking images designed in Microsoft^®^ PowerPoint^®^ that consisted of white squares (3.2×3.2 cm) on a dark background. For the control homogenous gels, one-step preparation was performed by irradiating the entire area of the gels for variable exposure times. The surface elastic modulus around the soft and stiff boundary was measured by the microindentation test, as previously described (Kawano and Kidoaki, 2011). Force-indentation curves on each gel surface were obtained using an atomic force microscope (AFM) (Nano Wizard 4, JPK Instruments, Germany) using a silicon-nitride cantilever with a half-pyramidal tip and a nominal spring constant of 0.02 N/m. Young’s moduli of the gel surfaces were evaluated from the force-indentation curves by fitting to the Hertz model (Hertz, 1881; Radmacher *et al.*, 1995; Wu *et al.*, 1998). The surface elasticity of the control homogenous and stiffness-gradient gels was measured from at least 10 randomly selected points and at 20 μm intervals from the stiff to soft boundary, respectively. The gel was stained with FITC-labeled albumin for an analysis of surface topography with a confocal microscope (LSM 510 Meta, Carl Zeiss, St. Louis, MO, USA), as previously described (Kuboki *et al.*, 2014).

### Measurement of cell trajectory, migration velocity, and cell shape

Cell trajectory, migration velocity and cell shape were measured with a time-lapse observation of the cells cultured on the gel substrates. The cells were allowed to adhere to the gel surfaces for 4–6 h and phase contrast time-lapse observation was performed using a 10X objective lens with a BZ-X700 (Keyence Corporation, Osaka, Japan). The movies were taken for 22 h at 15-min intervals. The cell trajectories were obtained by manually tracking the center of nuclei of the moving cells using the MTrackJ plugin in Fiji (https://fiji.sc/). The cell shapes were manually traced for the edges of cells using Fiji. Mean velocity and migration index were calculated as previously described (Kidoaki and Matsuda, 2008). Mean path velocity is defined as total moving distance along cell trajectory divided by total observation time. The total observation time for calculation was fixed at 3 hours. Migration index (MI) is defined as the ratio of net translocation distance (NTD) between starting and ending position to total moving distance (TMD), which denotes the magnitude of persistence of the cell migration; MI=1 indicates that the cell shows straight motion.

### Quantification of cell polarity at the elasticity boundary

Cells that moved from the soft region toward the stiff domain of the surface elasticity boundary were selected for shape analysis based on our previous work (Ebata *et al.*, 2018). The distance *R*(*θ*) from the centroid to the cell edge was calculated as a function of the angle *θ*, where *θ* is measured from the *x* axis. Next, complex Fourier coefficient *C_n_*(*t*) was calculated from *R*(*θ*, *t*). The complex Fourier coefficient *C_n_*(*t*) of the spatiotemporal shape *R*(*θ*, *t*) is defined as

$$R=R_{0}+\sum_{n=2}^{m}\left(C_{n}\mathrm{(t)}e^{in\theta }+C_{-n}\mathrm{(t)}e^{-in\theta }\right),$$

where *R_0_* is the mean radius and *m* is the number of data points. *C-_n_* is the complex conjugate of *C_n_*. The amplitude |*C_n_*(*t*)| corresponds to the magnitude of deformation, and the phase *φ_n_* represents the direction of maximum deformation, where *φ_n_* is defined as *C_n_*=|*C_n_*| exp *in* *φ_n_*. To quantitatively characterize the asymmetric deformation of cell shape that gives a typical direction, like an arrow or boomerang shape, *C-_2_C_3_* is useful because it satisfies a necessary condition of polarity of cell shape (Ohta *et al.*, 2016). From a symmetry argument, the polarity of the shape should have the same symmetry as the velocity; they are invariant only under a 360-degree rotation of the coordinates. Since mode n=1, *C_1_*, is included in the velocity, only the nonlinear terms of *C_n_* can satisfy the requirement. In the case of the second nonlinear terms, *C-_n_C_n+1_* satisfies symmetry. In this study, we used *C-_2_C_3_* as the cell polarity parameter.

### FRAP analysis

FRAP analysis at the leading edges of Venus-paxillin-expressing 3T3s was performed using a 488 nm Argon laser of a confocal microscope (LSM Meta 510) based on the optimization of bleaching conditions previously described (Axelrod *et al.*, 1976; Snapp *et al.*, 2003; Phair *et al.*, 2004; Carisey *et al.*, 2011). The cells were cultured on gels with either homogenous elasticity (25 kPa, 40 kPa, 150 kPa or 250 kPa) or square stiff domains (stiff domain 300 kPa/ soft base 35 kPa) overnight before analysis. Scanning was performed with a 60X objective lens by setting a pinhole at 5 Airy units with 8X optical zoom and 2-line averaging of 512 X 512 pixel-resolution images. The laser output was set at 45% and the laser transmission was set at 1% for scanning of pre- and post-bleaching images. During bleaching, the laser transmission was increased to 100%. The regions of interest (ROI) were set to cover the whole structure of the focal adhesion and bleaching was performed for 5 iterations (1.57 s). Pre-bleached scanning was performed for 10 images and post-bleached images were collected without a time interval until the fluorescent intensity reached a plateau. In each experiment, approximately 5–10 FAs from 3–5 cells were bleached. For FRAP analysis on gels with a square stiff domain, bleaching was performed for cells that were crossing the elasticity boundary, and for cells in the middle of the stiff domain. Durotactic cells at the elasticity boundary, for which the nucleus was located within the area of the elasticity transition and had leading edges approaching the stiff domain and trailing edges that were still on the soft base, were selected for FRAP analysis and defined as cells crossing the boundary.

### Statistical analysis

Non-parametric Mann–Whitney U test was performed for cell-shaping analysis and comparison of cell migration velocity between non-transfected and Venus-paxillin transfected cells. For the evaluation of cell migration velocity at elasticity boundary and FRAP analysis, non-parametric Kruskal Wallis test followed by Steel Dwass post-hoc test was conducted. All the data were obtained from at least 3 independent experiments and expressed as the mean±SD (standard deviation).

## Results

### Characterization of an elasticity boundary to examine durotaxis

**Fig. 1:**
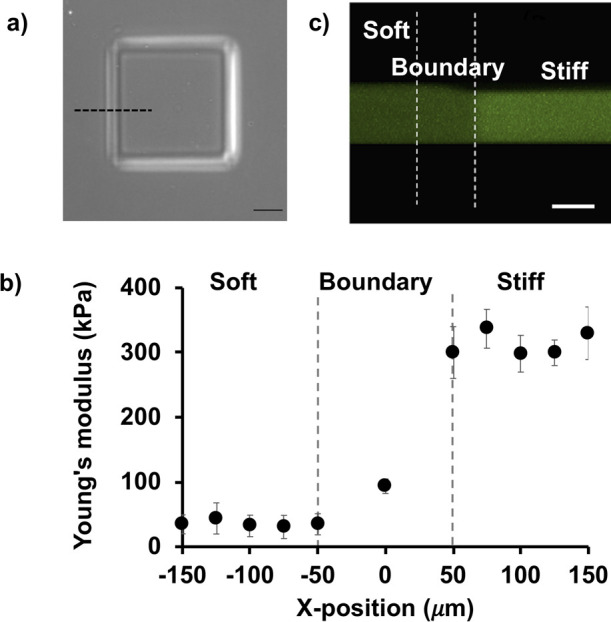
Elasticity boundary for investigating durotactic cells. a) Phase contrast image of the square stiff domain gel on the soft base. The scale bar is 100 μm. b) Elasticity distribution measured along the elasticity boundary indicated by the broken line in a). c) Cross-sectional CLSM image of the elastically-patterned gel across the boundary. The scale bar is 50 μm.

To induce and investigate durotactic cell movement, stiffness gradient and surface topography at the elasticity boundary were designed on microelastically-patterned gels with a 400×400 μm square stiff domain (Fig. 1a). Young’s moduli of soft and stiff regions were prepared as 35 kPa and 300 kPa, respectively (Fig. 1b). Here, the elasticity boundary was defined as the region between edges of elastically-flat plain in soft and stiff regions. The center of boundary region was set as zero position of the boundary, which is exactly the 200 μm-distant position from the center of stiff domain. The stiffness gradient was set to be ca. 300 kPa/50 μm, which was intentionally adopted weaker than a previously reported gradient that could induce strong durotaxis in 3T3 fibroblast cells, i.e., 400 kPa/50 μm on gelatin gels (Kawano and Kidoaki, 2011). Though the present setting of magnitude of Young’s modulus in stiff region was higher than that of most living tissues, it is the natural outcome of stiffness-setting for soft base region. We have previously clarified that the threshold stiffness gradient to induce durotaxis varies with depending on the absolute stiffness in soft region (Moriyama and Kidoaki, 2019). For example, if the elastic modulus of soft region is set lower than 2.5 kPa, threshold elastic modulus of stiff region is about 15 kPa. While this condition is near to physiological situation, the speed of durotaxis around the elasticity boundary becomes too high to catch the moment of cell crossing the boundary due to strong tendency escaping from very soft region. Therefore, in this study, 300/35 kPa condition was set in order to slow the speed of migration to focus on tracking of the dynamic behaviors of durotactic cells. With regard to the surface topography at the elasticity boundary, while the soft region was slightly swollen (Fig. 1c), the transition between the soft and stiff regions was smooth so as not to disturb natural durotactic migration, as confirmed previously (Ueki and Kidoaki, 2015).

### Durotactic migration of Venus-paxillin-exressing 3T3 cells at an elasticity boundary

**Fig. 2:**
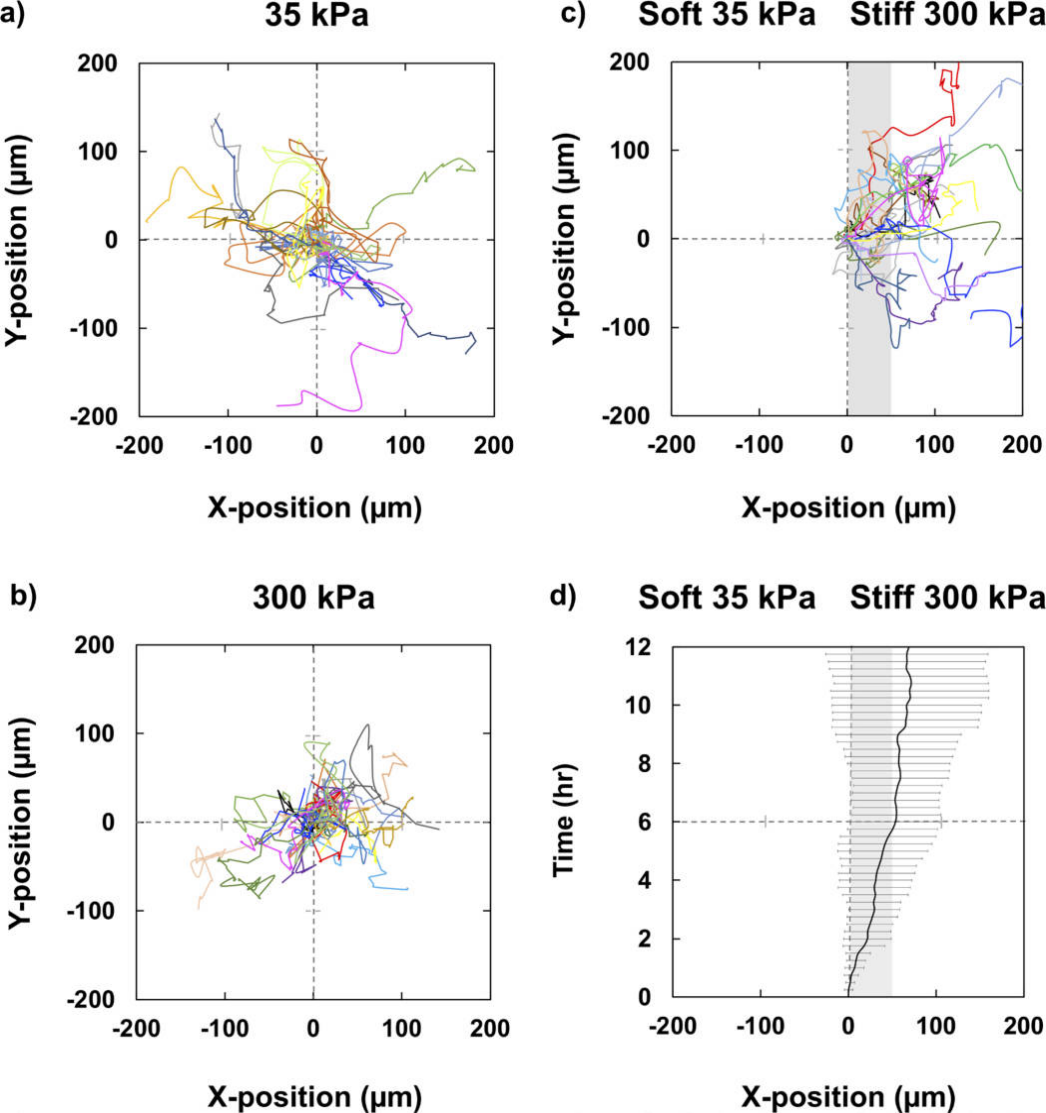
Random and durotactic migration of Venus-paxillin expressing 3T3s moving on surface-elasticity hydrogels. Superimposed cell trajectories a) on a 300 kPa homogeneous stiff control gel (N=16), b) on a 35 kPa homogeneous soft control gel (N=20), and c) around the elasticity boundary (N=18). The starting points of each cell were set into the origin of the graph. In c), all of the whole trajectories were rotated so that the right side of graph shows stiff region. As for the precise definition of Y axis, see the text. d) The ensemble-averaged X-trajectories calculated from c) with standard deviation. Shaded area in c) and d) indicate the range of elasticity boundary region.

The trajectories of cells stably expressing the fusion protein of paxillin with the yellow fluorescent protein Venus (Venus-paxillin) were measured by time-lapse observation on the homogenous control gels and at the elasticity boundary of the pattern gels. Fig. 2a and b show the typical migration behavior of cells on gel substrates with homogeneous distribution of Young’s moduli of 35 and 300 kPa, respectively. The motility of the cells on the stiff 300 kPa gel was suppressed in comparison to that on the softer 35 kPa gel. In Fig. 2c, all of the starting positions of raw cell trajectories around stiff domains (Fig. S1) were superimposed into the origin of the graph and all of the whole trajectories were rotated so that the right side of graph shows stiff region. Here, the “starting position” of each cell trajectory means the position where measurement of the trajectory was started, which was chosen from the cells located within 50 μm-distant region from the center of elasticity boundary region defined above. Therefore, ‘0’ in X-position or Y-axis indicates the starting positions of each cell trajectory, and real centers of elasticity boundary region distributes within 50 μm-distant right region from the Y-axis. The averaged X positions and the standard deviations of the cell trajectories were plotted against time as shown in Fig. 2d, which clearly shows markedly biased migration from soft to stiff region and reverse movement from stiff to soft region is very minor as indicated from small overlapping of the standard deviation in left-sided soft region even in the long culture period over 8 hrs.. The results clearly demonstrated preferential migration toward the stiff region, i.e., durotaxis was induced.

The migration behaviors of the untransfected 3T3 cells were also investigated on the homogeneous soft, stiff and patterned gels (supplementary information). No significant difference in the velocity of both random and durotactic motile cells was observed in comparison to that of the Venus-paxillin transfected cells. In comparison with the random motile cells, significant enhancement of cells migration velocities was noticeable in durotactic cells (Fig. S2).

**Fig. 3:**
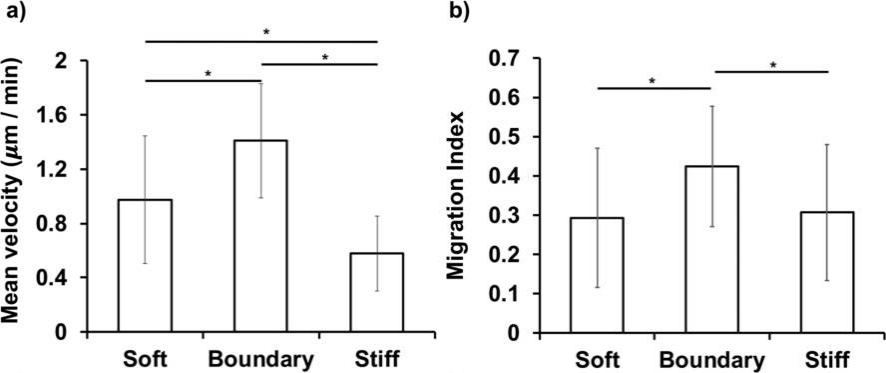
Characterization of durotactic properties. Cell Migration velocity on the soft region, elasticity boundary and on the stiff domain. The mean path velocity and migration index (MI) were measured for cells on the soft region (soft, N=35), and the nuclei of which had crossed the elasticity boundary (boundary, N=27), and for cells that were completely within the stiff domain (stiff, N=37). Statistically significant difference between groups was determined by Statistical significance (* *p*<0.05) was determined by Kruskal Wallis followed by Steel Dwass post-hoc tests.

The mean velocity of the cells at the elasticity boundary is shown in Fig. 3a. The cells in the soft region were moved at an average speed of about 0.97±0.47 μm /min. The cells appeared to accelerate to 1.40±0.42 μm /min at the elasticity boundary. After cells entered the stiff domain, the average speed decreased to 0.57±0.27 μm /min. The migration index (MI) of the cells was significantly higher than that of on the soft base and stiff domain (Fig. 3b). Representative contours of cells moving on homogenous gels with different elasticities are shown in Fig. S3a (supplementary movies M1–M4). The cells showed non-oriented and non-directional movement within one hour for each condition. On the other hand, cells at the boundary of the domain-patterned gels were highly motile and significantly migrated rapidly over a large distance across the boundary (Fig. S3b, supplementary movie M5). Most of the cells studied exhibited polarization, with the clear formation of leading and trailing edges upon reaching the elasticity boundary.

### Polarization of durotactic cells

**Fig. 4:**
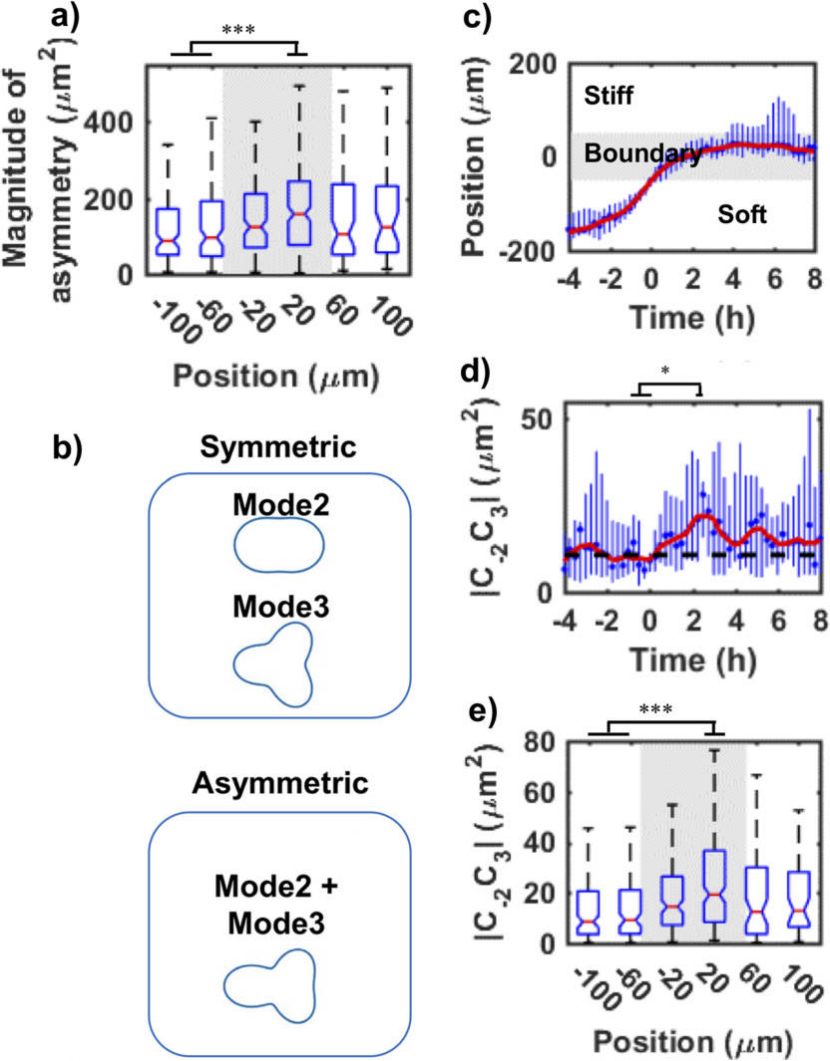
Cell-shaping dynamics of durotactic cells. a) Dependence of the magnitude of front-rear asymmetry of the cell shape on the cell position. A negative position indicates that the cell centroid was in the soft region. The gray-shaded area represents the boundary region. b) Schematic illustration of symmetric and asymmetric deformations of cell-shaping that can be described by the combination of Fourier modes. c) and d) Time evolution of cell trajectories and polarity parameter |*C-_2_C_3_*|, respectively. We set time T=0 when the cells first touch the boundary. T<0 indicates that the whole cell stays in the soft region. Dot symbols represent the median of the data from 20 cells. Bars connect the 0.25 and 0.75 quartiles of the data. Red curves show the smoothed curves of dot symbols. Black dashed lines denote reference values in the soft region, which were calculated by averaging the polarities from T=–4 h to 0 h. e) Position-dependent change in polarity parameter |*C-_2_C_3_*|. A negative position indicates that the cell centroid was in the soft region. The gray-shaded area represents the boundary region. a) and e) The edges of the box denote the 0.25 and 0.75 quartiles. The whiskers extend to the minimum and maximum data points without outliers. Note that outliers are not plotted here, because the distribution of the data has long tails. a), d) and e) The Mann–Whitney U test was used to calculate the *p* value. * *p*<0.05. *** *p*<0.001. a)–e) N=20.

To characterize the kinetic process of development of durotactic polarity, cell-shaping dynamics was first analyzed in terms of cell-scale front-rear polarization. Crawling cells typically have asymmetric shape with well-spread anterior part and narrow posterior part. As we previously reported, the durotactic cell just crossing the elasticity boundary certainly exhibits rich amount of mature FAs in anterior part entering in the stiff region and poor amount of FAs in the posterior part standing in the soft region (Kawano and Kidoaki, 2011). Thus, to evaluate the front-rear polarization, simple quantification of front-rear asymmetry of the cell shaping should be effective as the intuitive approach. The magnitude of front-rear asymmetry was calculated around the elasticity boundary region with respect to the short and long axes of elongated cell body (see Supplementary Method and Fig. S4) as a function of the cell position, which significantly increased in the boundary region (Fig. 4a).

On the other hand, the above analysis is valid only when the cell has elongated shape; it cannot adequately deal with less elongated shape like equilateral triangle that should appear in the process of development of motile polarity. Thus, we introduced more general analytical method by using the complex Fourier coefficient *C_n_* of cell-shape *R* (PRD model, see Method (Ebata *et al.*, 2018)). *C_n_* denote the magnitude and direction of the polygonal components of the cell shape: e.g., *C_2_*, *C_3_*, and *C_4_* represent elliptical, triangular, and quadrilateral deformations, respectively. We defined *C-_2_C_3_* as the polarity parameter of the cell shape, where *C-_2_* is a complex conjugate of *C_2_* (see Method for details (Ohta *et al.*, 2016)). *C-_2_C_3_* characterizes an asymmetric shape of an elongated triangle. For example, as shown in Fig. 4b, for pure elongated (mode2) and equilateral triangular (mode3) cells, shapes are symmetric and show no polarity. On the other hand, shape becomes asymmetric when the cells have both n=2 and n=3 Fourier modes (mode2+mode3), which is often seen for durotactic cells. The former symmetric shape gives *C-_2_C_3_*=0, while the latter asymmetric shape satisfies *C-_2_C_3_*≠0. We confirmed that |*C-_2_C_3_*| had rather strong positive correlation with the magnitude of front-rear asymmetry of elongated cell body (see Fig. S4).

To connect durotactic movement with the dynamics of polarity, we analyzed the trajectories of 20 durotactic cells that migrated through the elasticity boundary from the soft region to the stiff region. Fig. 4c shows the time course of the distance between the cell centroid and the center of the elasticity boundary region, which illustrates how cells approach the stiff region. The cell position is set to be negative when the cell is in the soft region. In Fig. 4c–d, for all cells, we set T=0 h when the leading edge of the cell touches the elasticity boundary. We then calculated the median of the data at each T. At the initial stage, when cells enter the elasticity boundary (gray area in Fig. 4c), the cells rapidly migrate toward the stiff region. As the cells approach the interior of the boundary region, they start to show gradual migration. A reason for this slower approach to the stiff region is that the cells tend to move along the boundary. Here, it should be noted that the slope of the plot in Fig. 4c represents only normal component of velocity against boundary, which does not correspond to the velocity along the trajectory path shown in Fig. 3 but qualitatively reflect the degree of change in moving direction. As this velocity component was normalized to the boundary, increasing slope in the soft region therefore means enhancement of directional movement of the cells toward the elasticity boundary.

Next, to evaluate the response of cell polarity to the elasticity gradient, we calculated the time course of polarity parameter |*C-_2_C_3_*| (Fig. 4d). Black dashed lines in Fig. 4d indicate the reference values in the soft region, which were calculated by averaging the values from T=–4 h to 0 h. |*C-_2_C_3_*| starts to increase when the cells touch the elasticity boundary (T=0 h). |*C-_2_C_3_*| has a peak at around T=2.5 h. This generation of polarity proceeds simultaneously with fast durotactic movement from T=0–1 h (Fig. 4c). After the peak, |*C-_2_C_3_*| gradually decreases to the reference values. The relaxation of polarity occurs in an oscillatory manner because the cells migrate with repeated extension and contraction of their bodies. The polarity and speed at which the cells approach the stiff region simultaneously relax (T=2–8 h in Fig. 4c, d).

Thirdly, to obtain spatial information on the generation of polarity, we explored the response of polarity in terms of cell position. Fig. 4e shows the dependence of |*C-_2_C_3_*| on the distance from the cell centroid to the center of the boundary region, which increased transiently only in the boundary region. When the cells migrated deeper into the stiff region, |*C-_2_C_3_*| decreased. Compared to |*C-_2_C_3_*| in the soft region, those in the stiff region have slightly high, albeit insignificantly so, values. This is because the cells have greater |*C_2_*| and |*C_3_*| on stiffer substrates (Ebata *et al.*, 2018). These results are comparable to those regarding the time courses (Fig. 4d). Fig. 4d and e suggest that the stiffness gradient at the boundary induces cell scale polarity in ca. 2.5 hr, and finally enhances directed migration. The representative snapshots of the cell that moved from the soft area, crossed the boundary and moved toward the stiff domain is shown in Fig. S5.

### Mobility of paxillin at focal adhesions in durotactic cells

To characterize the asymmetric activity of FAs in anterior and posterior part of durotactic cells, live-cell imaging of Venus-paxillin in FAs and FRAP analysis were performed. The entire areas of FA were bleached and fluorescence recovery curves were obtained. The FRAP measurements were performed only for the FAs in the anterior part of the moving cells due to the experimental difficulty on the FRAP measurements for the FAs in trailing edges of the cell. Since durotactic cell moves so fast around the elasticity boundary as shown in Fig. 3a, live time of trailing edge was inevitably short. To catch the moment of a durotactic cell just crossing the elasticity boundary was rather rare in the total time-lapse observation, which tended to become more difficult in the case of FRAP experiment for such a rare event of a cell. From these real experimental difficulties, capturing enough number of measurements for FA dynamics in trailing edges was abandoned in this study, but the front-rear asymmetricity of FA dynamics was characterized with another devised methodology as described below.

**Fig. 5:**
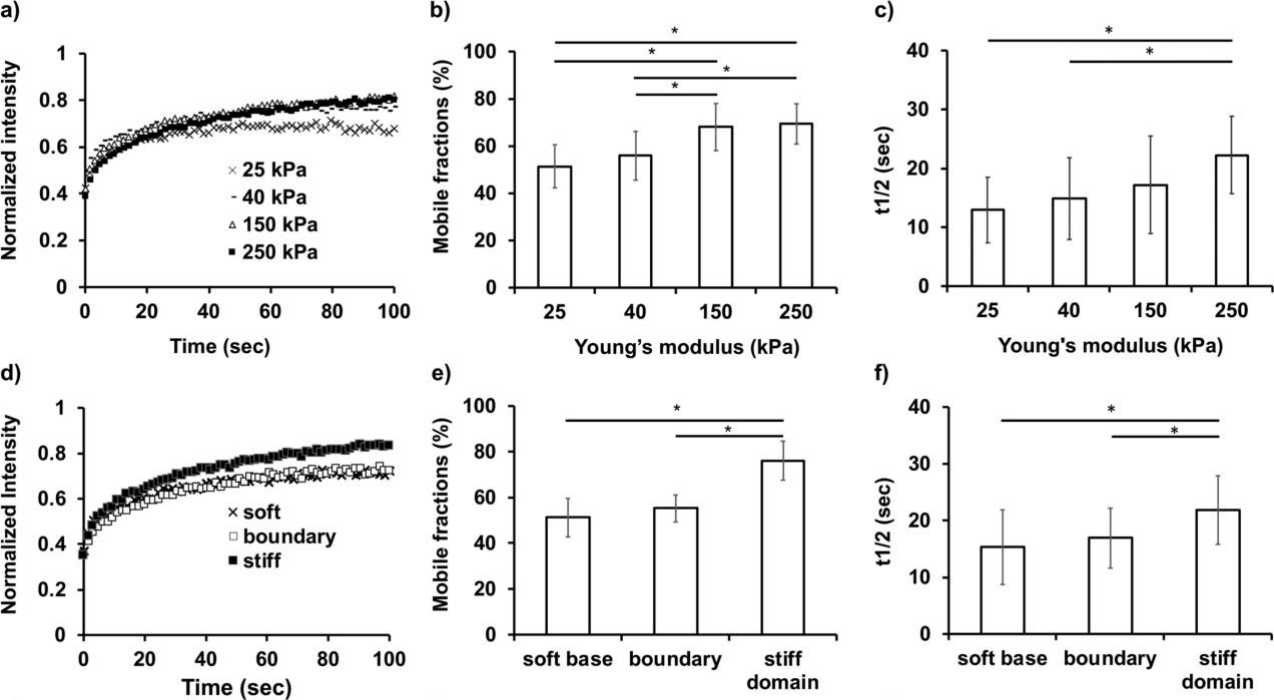
FRAP analysis of paxillin mobility of durotactic cells. a) Averaged fluorescence recovery curves of Venus-paxillin on 25, 40, 150 and 250 kPa control gels. b) Mobile fractions of paxillin calculated for gels of 25, 40, 150 and 250 kPa and c) the corresponding t_1/2_. d) The averaged fluorescence recovery curves of Venus-paxillin on the soft, border and hard regions of domain-patterned gels. e) Mobile fractions of paxillin calculated around the elasticity boundary of domain-patterned gels and f) the corresponding t_1/2._ The error bars represent standard deviations. A total of 30–40 FAs from 5 independent experiments were analyzed. Statistical significance (* *p*<0.05) was determined by Kruskal Wallis followed by Steel Dwass post-hoc tests.

The recovery curves were used to calculate the mobile fractions of paxillin and half-time of fluorescent recovery (t_1/2_) was calculated from the curves fitting to the single exponential function (Axelrod *et al.*, 1976; Snapp *et al.*, 2003; Phair *et al.*, 2004; Carisey *et al.*, 2011). Fig. 5a shows the recovery curves on control homogenous gels with various stiffness values (Representative FRAP images are shown in Fig. S6). The data revealed that paxillin diffusion was stiffness-dependent, since the percentage of fluorescence recovery increased with gel stiffness. The results suggested that the mobile fractions of paxillin increased (Fig. 5b) and t_1/2_ also slightly increased (Fig. 5c) when the stiffness of the gels increased.

FRAP analysis of paxillin dynamics for the anterior part of durotactic cells was performed in the soft base region, the elasticity boundary, and the middle of the stiff domain (Fig. 5d–f and Fig. S7). In agreement with the results with control homogenous gels, the cells in the soft region exhibited lower mobile fractions of paxillin at FA than those in the stiff domain. Interestingly, cells in the boundary with their front edges spread in the stiff domain still kept similar fractions on paxillin mobility to those in the soft region. After the cells completely entered the stiff domain, the mobile fractions clearly increased. This suggested that the diffusion mobility of paxillin in the anterior part of durotactic cells gradually switched from a retarded immobile mode at the elasticity boundary to a higher mobile mode after the cells moved inside the stiff region.

**Fig. 6:**
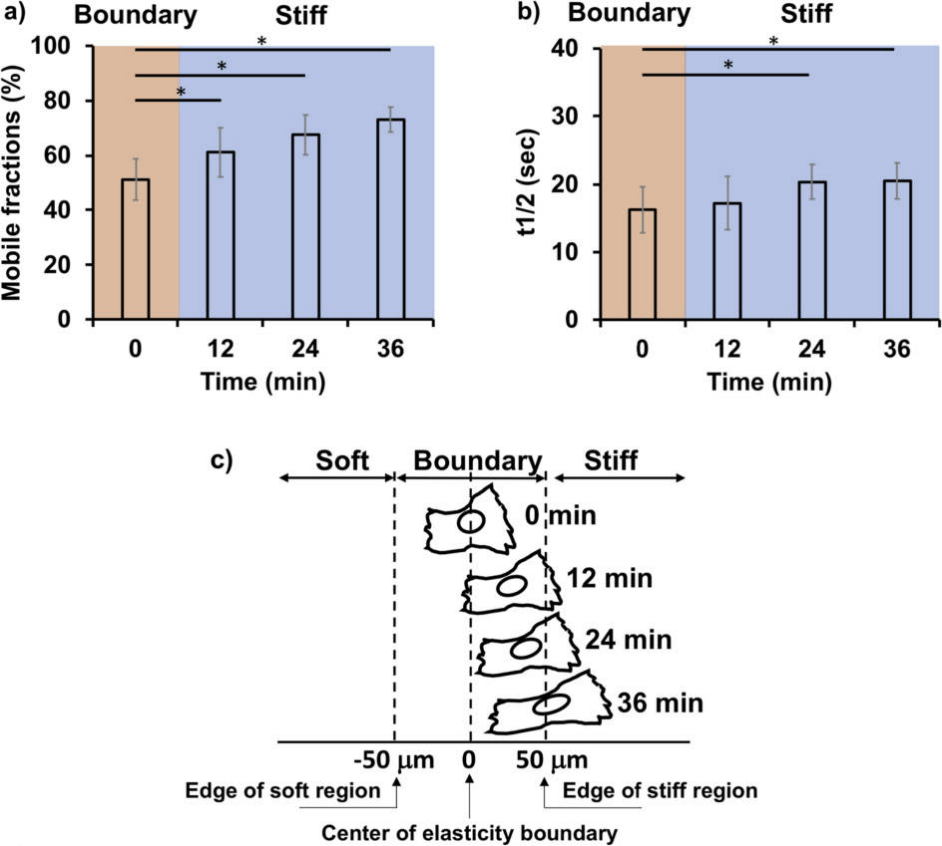
Time-dependent mobility change of paxillin around the elasticity boundary. a) Mobile fractions of Venus-paxillin of the durotactic cells just crossing the elasticity boundary and b) the corresponding t_1/2_. Cells whose nucleus is located at the center of elasticity boundary (T=0) and just approaching to the stiff region were selected for FRAP analysis as schematically shown in c). The FRAP analysis was sequentially performed on the same cells at 12, 24 and 36 min inside the stiff region. A total of 30–40 FAs from 5 independent experiments were analyzed. The error bars represent standard deviations. Statistical significance (* *p*<0.05) was determined by Kruskal Wallis followed by Steel Dwass post-hoc tests.

The time-dependent mobility of paxillin at FA was further studied by tracking diffusion in terms of the mobile fractions within the same cells that migrated across the elasticity boundary region (gray area of Fig. 4c). The first fluorescence recovery curve, that was obtained for FAs in the anterior part of a cell whose nucleus is located at the center of elasticity boundary, was arbitrarily defined as time 0 and the fractions on mobility of paxillin were plotted against time at 12-min intervals (Fig. 6a) with the corresponding t_1/2_ (Fig. 6b). It should be noted that turnover of paxillin in the FAs in anterior part of a cell was significantly faster in the elasticity boundary region than in the stiff region (Fig. 5f), which can be statistically confirmed after 12 min (Fig. 6a and b). This means that the leading edge of the cells moved across the stiff-sided edge of elasticity boundary between ca.10 and 20 min as schematically shown in Fig. 6c. In this situation, front-rear asymmetricity in terms of dynamics of FAs was found to be established within ca. 20 min across the stiff-sided edge of the elasticity boundary, which markedly leaded up to the 2.5-hr of cell-scale polarity generation characterized above.

## Discussion

In general, cell movements are the result of various hierarchical molecular processes on different spatiotemporal scales. For example, cells start to move after adhering to ECM or the substrate surface, the former of which involves the nanometer-scale molecular bindings between integrin and its ligand of adhesive proteins with micro- to millisecond-order kinetics (Ridley *et al.*, 2003), followed by the micrometer-scale assembly of many kinds of proteins to form FAs with several tens of minutes-order kinetics (Gardel *et al.*, 2010). The mechanical interactions between ECM and intracellular cytoskeletons (CSKs) via multiple FAs distributed over the entire adhesion interface of a single cell determine the cell shape, and the cell-shaping dynamics regulate the direction of cell movement with hour-order kinetics (Ladoux *et al.*, 2016). In this sense, the mechanism of directional movement of adherent cells is governed in principle by different hierarchical molecular processes with different time-scales. To understand the mechanism, the relationships among these multiscale spatiotemporal kinetics should be addressed. An effective approach to investigating these relationships is to focus on the emergence of cell polarity in the surface-dependent tactic movement. Especially, durotactic cells crossing an elasticity boundary should exhibit the hierarchical kinetic process in cell polarity generation in a well-organized order at the point of crossing. In this study, by using a model substrate with a precise surface stiffness gradient, cell-shape polarity and turnover activity of paxillin in FAs were characterized for durotactic cells just crossing the elasticity boundary with respect to the timing of the emergence and establishment of cellular motile polarity.

First, we clarified how an elasticity boundary could induce asymmetric changes in polarity at a cell-body scale between the anterior and posterior parts. From the Fourier mode analysis, the cell-shape polarization was established in ca. 2.5 hr at the elasticity boundary where acto-myosin filament-generated force rapidly increase as cells coming from the soft part (Trichet *et al.*, 2012), and durotactic cells could generate asymmetrical strains and develop stronger traction forces for the stiffer region (Breckenridge *et al.*, 2014). This observation indicates that an asymmetric distribution of traction-forces and strains contribute to symmetry-breaking of the durotactic cells, and the exact timescale is several-hour-order.

Next, we tried to gain insight into the asymmetric dynamics of paxillin in FAs between anterior and posterior parts across the boundary. The mobility of paxillin localized in the anterior part gradually increased at the elasticity boundary as the cells approached the stiff region. The increase in the dissociation of paxillin in FAs was also time-dependent during the crossing processes, i.e., as the duration that cells experienced the stiff substrate increased, the mobile fraction of paxillin also increased, indicating that the binding interaction of paxillin with its partners decreased when durotactic cells crossed the elasticity boundary. Several lines of evidence have suggested that the interaction of paxillin with its binding partners is responsible for cell polarization during directional cell migration (Nishiya *et al.*, 2005; Yu *et al.*, 2009). In a recent study, several FA proteins were categorized into different modules based on their mobilities and functions (Stutchbury *et al.*, 2017). Structural module proteins that are located in the force transduction layer in FA complex, such as talin and vinculin (Kanchanawong *et al.*, 2010), turnover slowly and are directly involved in rigidity-sensing. On the other hand, paxillin and FAK, which are signaling module proteins that are compartmentalized in the integrin signaling layer in FA complex, turnover very quickly, and therefore potentially reside transiently in FAs. The observed transition of paxillin mobility at the elasticity boundary indicates that the gradient of mechanical stimuli regulates the front-rear asymmetric activity of signaling module of FAs in durotactic cells, and the asymmetricity in FA-scale is established within 30 min.

**Fig. 7:**
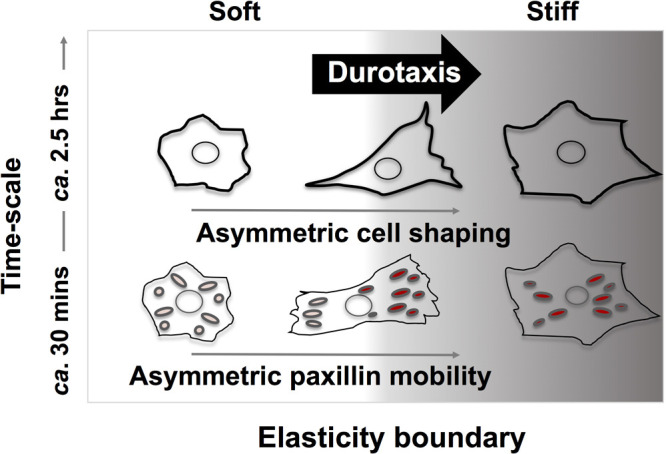
Schematic representation of hierarchical step-wise development of motile polarity in durotactic cells. The asymmetric mechanical stimuli from the stiffness gradient induce the asymmetricity in FA dynamics across the boundary within thirty minutes. The subsequent establishment of asymmetricity in cell-shaping occur ca. two and a half hours later.

Conclusively considering the observed time-scale of asymmetricity generation in cell-shaping and FA activity, FA-scale asymmetricity appears in ca. 30 min then cell-shaping asymmetricity are retarded to establish in ca. 2.5 hr for durotactic cells just crossing the elasticity boundary. Several studies have reported the sequences of the regulation of FA dynamics, cell shape and migratory behaviors (Vasiliev, 1985; Xia *et al.*, 2008; Prager-Khoutorsky *et al.*, 2011; Kim and Wirtz, 2013). FA alignment tended to precede the overall elongation of cells, indicating that FA orientation may direct cell polarization (Prager-Khoutorsky *et al.*, 2011). Our *in situ* observation of durotactic cells suggested that motile polarity of crawling cells develops in a hierarchical stepwise manner from the microscopic asymmetric response of FA dynamics to the alteration of cell-scale polarization with a several-hour time lag (Fig. 7). Stiffness- and spatiotemporal-dependent regulation of the interaction between paxillin and its binding partners in correlation with asymmetric shape fluctuations are emphasized as contributing factors to induce durotaxis.

## Acknowledgments

This work was financially supported by the Advanced Research and Development Programs for Medical Innovation from the Japan Agency of Medical Research and Development (AMED-CREST, JP19gm0810002) and the Dynamic Alliance for Open Innovation Bridging Human, Environment and Materials (Five-star Alliance).

## Additional information

Competing interests: The authors declare no competing interests.

## Abbreviations

StG: styrenated gelatin
AFM: atomic force microscope
FAs: focal adhesions
PRD: persistent random deformation
FRAP: fluorescent recovery after photobleaching
