# Supplementary material for: Hierarchical Development of Motile Polarity in Durotactic Cells Just Crossing an Elasticity Boundary
Source: Cell Struct Funct. 2019 Dec 27;45(1):33–43. doi: 10.1247/csf.19040 (PMC10739161; doi:10.1247/csf.19040)
Supplement: Supplementary file 1 — Supplementary information [file csf_45_19040_1.pdf]

Hierarchical development of motile polarity in durotactic cells just crossing an elasticity boundary

Thasaneeya Kuboki[1], Hiroyuki Ebata [1], Tomoki Matsuda[2], Yoshiyuki Arai[2], Takeharu Nagai[2] and Satoru Kidoaki [1]*

1. Laboratory of Biomedical and Biophysical Chemistry, Institute for Materials Chemistry and Engineering, Kyushu University, 744 Moto-oka, Nishi ku, Fukuoka, Japan

2. Department of Biomolecular Science and Engineering. The Institute of Scientific and Industrial Research, Osaka University, Mihogaoka 8-1, Ibaraki, Osaka, Japan

* Corresponding Author: kidoaki@ms.ifoc.kyushu-u.ac.jp; tel.: 81-92-802-2507; fax: 81-92-802-2509

Supplementary methods

Cloning of Venus-paxillin

E. coli DH5 alpha harboring pEF-1α plasmid vector was used for plasmid preparation with a Qiagen plasmid mini kit (Qiagen, Tokyo, Japan). Five micrograms of the pEF-1α plasmid was digested with *Eco*RI at 37°C for 1.5 h and further treated with calf intestinal phosphatase for 1 h at 50°C. The digested product was separated by agarose gel electrophoresis and the linearized vector band was excised from the gel and purified by a Geneclean II kit (MP Biomedical, Tokyo, Japan), according to the manufacturer's instructions. The gene encoding the yellow fluorescent protein Venus was amplified by PCR using primers flanked by *Eco*RI restriction sites, the modified Kozak sequences and start codon (forward primer 5' GGC*GAATTC***ACC**ATG**G**TGAGCAAGGGC 3', reverse primer 5' GGC*GAATTC*CTTGTACAGCTCGTC 3'). The amplified product was digested with *Eco*RI and purified by Geneclean. The purified product was used for

ligation with the prepared vector using a DNA Ligation Kit, Mighty Mix (Takara, Tokyo, Japan). The ligated DNA was transformed into *E. coli* DH5 alpha and plated onto LB-ampicillin plates. Several colonies were randomly selected for PCR screening. Positive clones with the correct orientation were selected for plasmid preparation and sequencing.

Five micrograms of pEF1-venus was digested with *Eco*RV and further treated with calf intestinal phosphatase. The full-length paxillin (1674 bp) was obtained by reverse transcription and PCR from human mesenchymal stem cells (MSCs) with gene-specific primers. The gene product was then amplified using primers flanked by *Eco*RV restriction sites (forward primer 5' GCG*GATATC*CAGCTGCTGCTGCAGCTGCTACCATGGACGACCTCGAC GCCCTG 3'; reverse primer 5' GCG*GATATC*TCTAGCAGAAGAG CTTGAG 3') for cloning in-frame with the c-terminal of pEF1-venus. 6-Alanine amino acids were introduced into the forward primer to serve as a linker to facilitate correct folding of the fusion protein. Blunt-ended ligation of the prepared vector and insert was performed and positive colonies were selected for sequencing.

Time-lapse analysis of the untransfected 3T3s and Venus-Paxillin expressing 3T3s

Time-lapse analysis of the untransfected 3T3s and Venus-Paxillin expressing 3T3s was performed as previously described for the cell polarity quantification. The cells were seeded on the tissue culture dishes, homogeneous control soft (35 kPa), stiff (300 kPa) and domain-patterned gels. The center of nuclei of the moving cells were manually tracked for the calculation of migration velocity. For the cells at the elasticity transition, the motile velocity was calculated from the trajectories of the cells moving from the soft region toward the stiff domain.

Fluorescence time-lapse observation of moving cells was performed using a 40X objective lens with an Olympus IX-71 microscope equipped with an EMCCD camera (Evolve 512, Photometrics, Tucson, AZ, USA) and Z stage controller (KOHZU SC.200, Kozu Precision Co., Ltd, Kawasaki, Japan). Venus-paxillin expressing 3T3s were seeded on gels at a density of 1.5 $\times$ 10^3 cells/cm^2 and cultured overnight in DMEM media with 10% FBS. The media was changed to phenol red-free, CO_2-independent Leibovitz's L-15 media supplemented with 10% FBS and time-lapse movies were obtained using Z-stacking mode of the imaging software (Metamorph, Molecular Devices, Sunnyvale, CA, USA) for 3-6 h at 5-min intervals. For data processing, all of the Z-stacking images for each time point were orthogonally projected into a single image using Z-project and the sum slice function of Image J (NIH, Bethesda, MD, USA). The sum slices for each time point were combined and used to generate movies.

Analysis of front-rear asymmetry of the cell shape

The magnitude of front-rear asymmetry was calculated with respect to the short and long axes of elongated cell body (Fig. S4). Here, to calculate the short and long axes, we fitted segmented cell image by ellipsoid (Matlab software). In the case of elongated triangle like fibroblast, asymmetry with respect to the short axis denotes front-rear asymmetry of the shape. To quantify the front-rear asymmetry, we divided segmented cell image into front and rear regions by using short axis. Then, we defined that the difference of the area ΔAB between front and rear regions was magnitude of the front-rear asymmetry (Fig. S4). In the case of banana shape like keratocyte, asymmetry with respect to the long axis denotes front-rear asymmetry of the shape. Similar to the case of the elongated triangle, we calculated the difference of the two area ΔCD, which were

divided by long axis (Fig. S4). In general, cells have both asymmetry. Therefore, as shown in a following equation, we combined these two values.

$$\text{Front} - \text{rear asymmetry of cell shape} = \Delta \text{AB}\vec{\text{n}}_L + \Delta \text{CD}\vec{\text{n}}_S$$

where $\vec{\text{n}}_L$ and $\vec{\text{n}}_S$ are the unit vector parallel to the long and short axes of the cell shape. We confirmed that the magnitude of front-rear asymmetry of cell shape had rather strong positive correlation with $|C_{-2}C_3|$ defined in the main text (Fig. S4).

Supplementary movies

Supplementary movies M1-M4 show the time-lapse movies of the Venus-paxillin expressing 3T3s on 4, 15, 40 and 120 kPa homogeneous gels respectively. The movies were taken for one hour with 3 min interval. Supplementary movie M5 show the time-lapse movies of the 3T3s expressing Venus-paxillin at the elasticity boundary of the domain-patterned gels. The movies were taken for one hour with 3 min interval.
