# Supplementary material for: Hierarchical Development of Motile Polarity in Durotactic Cells Just Crossing an Elasticity Boundary
Source: Cell Struct Funct. 2019 Dec 27;45(1):33–43. doi: 10.1247/csf.19040 (PMC10739161; doi:10.1247/csf.19040)
Supplement: Supplementary file 7 — Fig. S1 [file csf_45_19040_7.pdf]

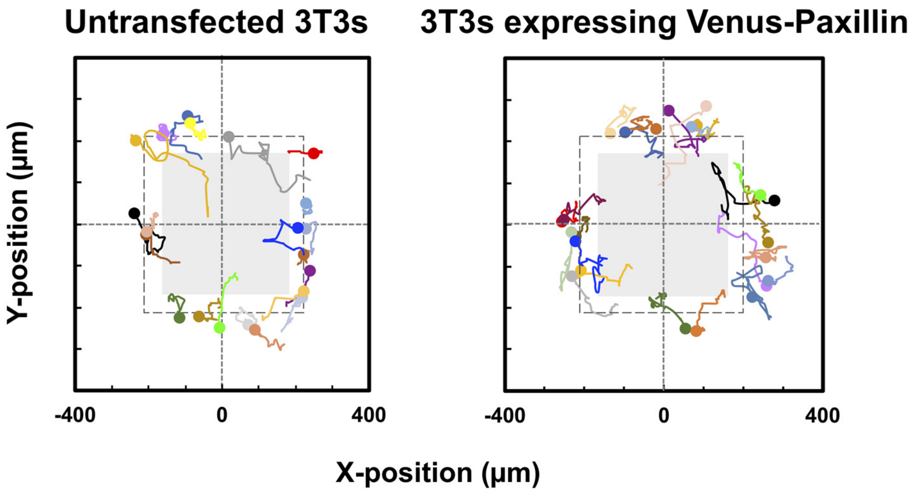

Figure S1. The trajectories of the untransfected and Venus-paxillin transfected 3T3s at the elasticity boundary. The durotactic behaviors of the untransfected 3T3s and Venus-paxillin expressing 3T3s were evaluated on the elasticity boundary of stiff square domain 300 kPa (gray area) on soft base 35 kPa. The broken lines indicated the center of elasticity boundary.
