# Supplementary material for: Hierarchical Development of Motile Polarity in Durotactic Cells Just Crossing an Elasticity Boundary
Source: Cell Struct Funct. 2019 Dec 27;45(1):33–43. doi: 10.1247/csf.19040 (PMC10739161; doi:10.1247/csf.19040)
Supplement: Supplementary file 8 — Fig. S2 [file csf_45_19040_8.pdf]

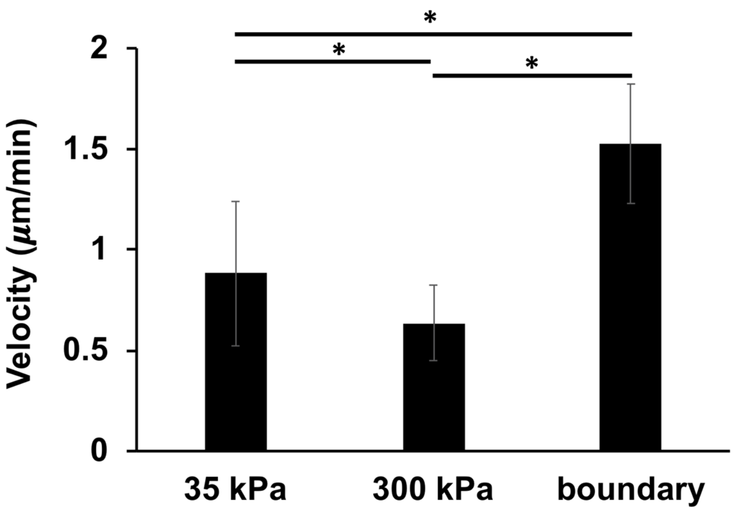

Figure S2. The motility of the Venus-paxillin transfected 3T3s on different surface elasticities. The migration velocity of the Venus-paxillin expressing 3T3s were evaluated on 35 kPa and 300 kPa homogenous gels and the elasticity boundary of stiff square domain 300 kPa on soft base 35 kPa. Analyses number of cells: on 35 kPa (N = 51), 300 kPa (N = 51), boundary (N = 26). Statistical significance (*$p < 0.05$) was determined by Kruskal Wallis followed by Steel Dwass.
