# Supplementary material for: Hierarchical Development of Motile Polarity in Durotactic Cells Just Crossing an Elasticity Boundary
Source: Cell Struct Funct. 2019 Dec 27;45(1):33–43. doi: 10.1247/csf.19040 (PMC10739161; doi:10.1247/csf.19040)
Supplement: Supplementary file 9 — Fig. S3 [file csf_45_19040_9.pdf]

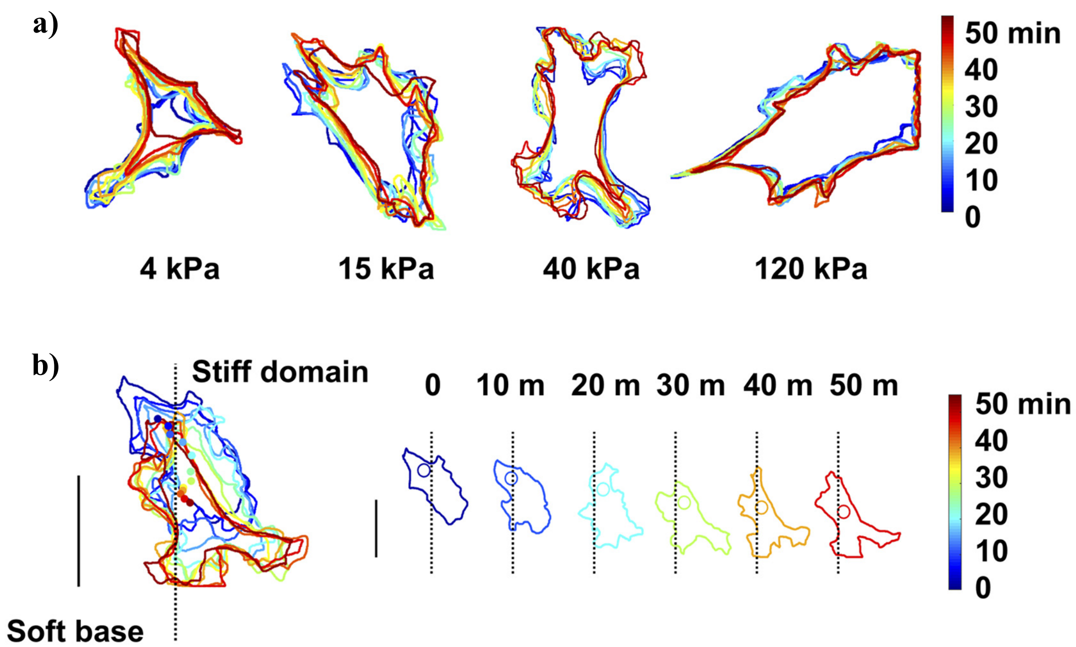

Figure S3. Morphological dynamics of random crawling cells and durotactic cells. Motile cells were extracted from time-lapse movies and overlaid in different colors. The total observation time was 50 min and observations were obtained at 5-min intervals. a) Random motile cells on homogeneous 4, 15, 40 and 120 kPa gels. b) Overlaid contours of directional motile cells moving from the soft region to the stiff domain. Images at 10-min intervals for durotactic cells just crossing the elasticity boundary (broken line). The scale bar is 50 µm. The color of the cell outlines was modified using the jet colormap algorithm of MATLAB software (Mathworks, Natick, MD, USA).
