# Supplementary material for: Hierarchical Development of Motile Polarity in Durotactic Cells Just Crossing an Elasticity Boundary
Source: Cell Struct Funct. 2019 Dec 27;45(1):33–43. doi: 10.1247/csf.19040 (PMC10739161; doi:10.1247/csf.19040)
Supplement: Supplementary file 10 — Fig. S4 [file csf_45_19040_10.pdf]

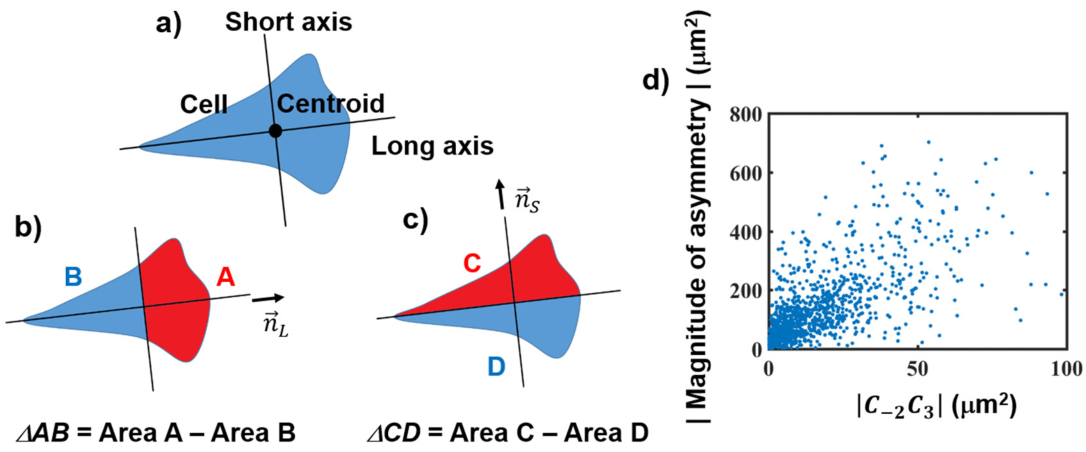

Figure S4. Analysis of front-rear asymmetry of the cell shape. a) Schematic illustration of the elongated cell. b) Magnitude of front-rear asymmetry ΔAB with respect to short axis. $\vec{n}_L$ represents unit vector parallel to the long axis. c) Magnitude of front-rear asymmetry ΔCD with respect to long axis. $\vec{n}_S$ represents unit vector parallel to the short axis. d) Correlation between magnitude of front-rear asymmetry of the shape and $|C_{-2}C_3|$. Correlation coefficient is 0.67.
