# Supplementary material for: Hierarchical Development of Motile Polarity in Durotactic Cells Just Crossing an Elasticity Boundary
Source: Cell Struct Funct. 2019 Dec 27;45(1):33–43. doi: 10.1247/csf.19040 (PMC10739161; doi:10.1247/csf.19040)
Supplement: Supplementary file 11 — Fig. S5 [file csf_45_19040_11.pdf]

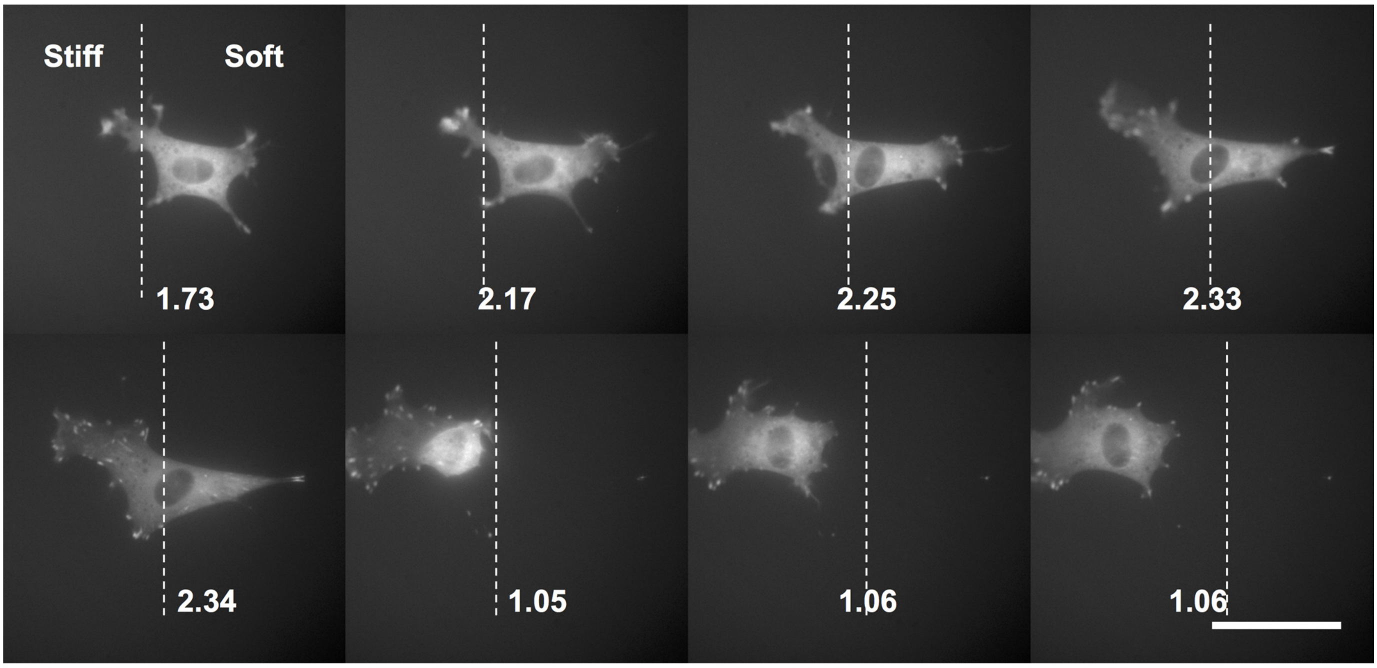

Figure S5. The typical shaping dynamics of the durotactic cells crossing elasticity boundary. The representative snapshots of the cells that move from the soft, cross the center of elasticity boundary (broken lines) and enter into the stiff region. The aspect ratio was calculated using Fiji. The corresponding aspect ratio clearly shows the increase in shape asymmetricity at the boundary region. The scale bar is 50 µm.
