# Supplementary material for: Hierarchical Development of Motile Polarity in Durotactic Cells Just Crossing an Elasticity Boundary
Source: Cell Struct Funct. 2019 Dec 27;45(1):33–43. doi: 10.1247/csf.19040 (PMC10739161; doi:10.1247/csf.19040)
Supplement: Supplementary file 12 — Fig. S6 [file csf_45_19040_12.pdf]

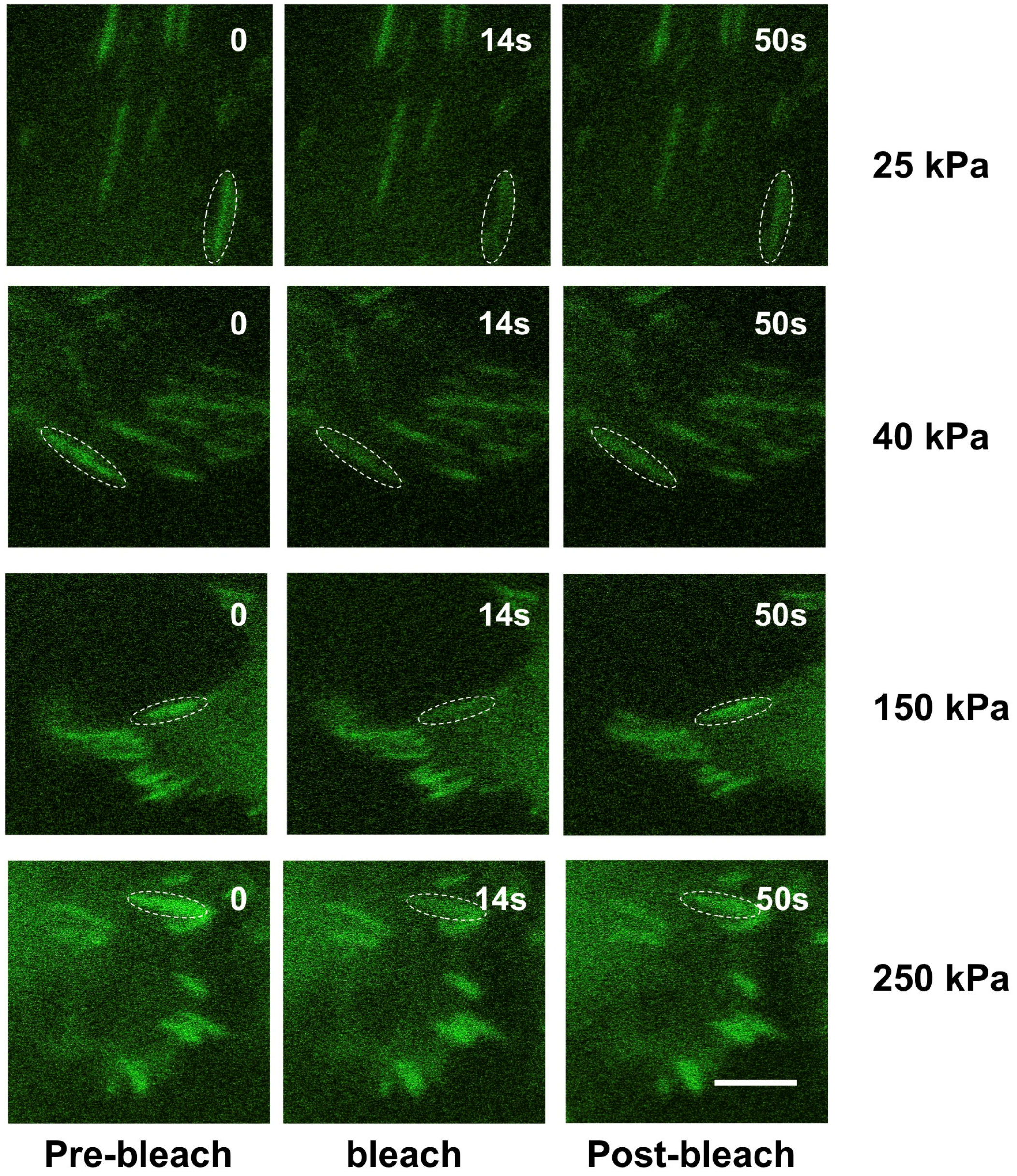

Figure S6. The confocal images of FRAP analysis on the 25, 40, 150 and 250 kPa homogeneous gels. The Region of Interest (ROI) of the focal adhesion before (pre-bleach), during (bleach) and after photo-bleaching (post-bleach) were indicated in the broken ellipsoidal lines. The scale bar is 5 μm
